# A longitudinal study on the impact of stroke patients’ perception trajectories of disease recurrence on short-term clinical outcomes

**DOI:** 10.3389/fneur.2026.1883000

**Published:** 2026-09-25

**Authors:** Weiwei Qian, Xiaoyan Sheng, Hui Zhang

**Affiliations:** 1 Department of Neurology, The Sixth People’s Hospital of Nantong, Nantong, Jiangsu, China; 2 Department of Neurology, Nanjing Gaochun People’s Hospital, Nanjing, Jiangsu, China

**Keywords:** latent class growth analysis, longitudinal study, prognosis, recurrence, risk perception, stroke, trajectory

## Abstract

**Objective:**

To investigate heterogeneous trajectories of recurrence risk perception among stroke patients within 6 months after discharge and their correlation with short-term clinical outcomes.

**Methods:**

A prospective observational study was conducted from February 2023 to October 2025 at the Sixth People’s Hospital of Nantong. Of 487 consecutive acute stroke patients screened, 262 met eligibility and provided informed consent, and 240 completed all four follow-ups and were included. Risk perception was assessed using the Stroke Recurrence Risk Assessment Scale at discharge and at 1, 3, and 6 months post-discharge. Clinical outcomes were evaluated using the modified Rankin Scale (mRS), NIHSS, Barthel Index (BI), and Stroke-Specific Quality of Life Scale (SS-QOL). Latent class growth analysis identified risk perception trajectories, and multivariable regression examined associations between trajectories and outcomes.

**Results:**

Four distinct risk perception trajectories were identified: the sustained low-risk perception group (35.0%, *n* = 84), the moderate-stable perception group (28.3%, *n* = 68), the delayed high-risk perception group (20.8%, *n* = 50), and the gradually declining perception group (15.9%, *n* = 38). Multivariable regression analysis, after adjusting for confounders, revealed that compared with the sustained low-risk perception group, the delayed high-risk perception group had a 2.85-fold higher odds of functional dependence at 6 months, an average NIHSS score 2.01 points higher, a Barthel Index score 12.31 points lower, and an SS-QOL total score 14.61 points lower (all *p* < 0.05). Regarding stroke recurrence, the delayed high-risk perception group had the highest recurrence rate (18.0%); however, given the small number of recurrence events during the 6-month follow-up (only 20 cases), this finding was treated as exploratory and descriptive only, and no multivariable regression analysis was performed. The other two trajectory groups showed no statistically significant differences compared with the reference group.

**Conclusion:**

Heterogeneous developmental trajectories of recurrence risk perception exist among stroke patients, and the “delayed high-risk perception” trajectory is significantly associated with adverse short-term clinical outcomes. Dynamic monitoring of changes in patients’ risk perception in clinical practice may help identify high-risk individuals and thus provide a reference for early psychological intervention.

## 1 Introduction

Stroke is one of the leading causes of death and long-term disability worldwide, imposing a heavy burden on healthcare systems, families, and society. A key factor exacerbating this burden is the high risk of stroke recurrence (1). Compared to a first-ever stroke, recurrent strokes are typically associated with worse neurological outcomes, higher mortality, and greater functional dependency (2). Although significant progress has been made in secondary prevention strategies, including optimized pharmacological therapy and risk factor management, stroke recurrence rates remain unacceptably high. The success of these strategies fundamentally depends on patients’ long-term adherence to health-protective behaviors, such as medication compliance, lifestyle modifications, and regular follow-up. Growing evidence indicates that these behaviors are profoundly influenced by patients’ perceptions and beliefs about their disease and treatment (3, 4). For instance, beliefs that medications are harmful or an underestimation of the severity of recurrence risk can directly lead to poor adherence. This aligns with the core concept of the ‘Health Belief Model’, wherein an individual’s perceived susceptibility to and perceived severity of a health threat are primary drivers of behavioral decision-making (5). Recent research further confirms that interventions designed based on this model can effectively improve patients’ control of risk factors and medication adherence (6). Therefore, for stroke survivors, the perception of recurrence risk—encompassing the personal assessment of the likelihood and anticipated severity of another stroke—is a critical psychological determinant that may either facilitate or hinder their engagement in long-term prevention.

Emerging research has begun to validate this association, indicating that stroke patients’ perception of recurrence risk is correlated with their self-management behaviors and emotional states (7). However, current understanding is hindered by a critical limitation: the vast majority of studies have employed cross-sectional designs, treating risk perception as a static trait measured at a single time point. The post-stroke period, particularly the first 6 months after the index event, is a dynamic phase of physical recovery, psychological adjustment, and identity re-negotiation (8). Both theory and clinical observation suggest that a patient’s sense of vulnerability is constantly evolving—it may intensify with the emergence of new symptoms, diminish with successful rehabilitation, or fluctuate with life events (9). Consequently, static measurements are unable to capture the potential heterogeneous trajectories of risk perception that may exist during this critical window. Understanding these distinct longitudinal patterns is crucial, as different trajectories may have differential impacts on clinical recovery and long-term adaptation.

Therefore, a significant knowledge gap exists. Evidence is lacking regarding: (1) what empirically derived longitudinal trajectories of stroke recurrence risk perception exist during the critical first 6 months after a stroke event; and (2) whether these dynamic psychological pathways are differentially associated with short-term objective clinical outcomes. Addressing this gap requires a longitudinal study design and analytical methods capable of identifying homogeneous subgroups that follow similar perceptual pathways over time. Accordingly, this prospective longitudinal cohort study aims to explore the dynamic relationship between psychological perception and clinical reality in the context of secondary stroke prevention. Its two primary objectives are: (1) to identify and characterize latent class trajectories of recurrence risk perception within 6 months post-stroke using a validated, stroke-specific instrument; and (2) to examine the independent associations between these perceptual trajectories and six-month clinical outcomes. By shifting the focus from static risk assessment to dynamic risk perception trajectories, this study seeks to generate new insights that may inform the future development of timely, individualized psychological interventions, ultimately contributing to improved mental health and clinical prognosis among stroke survivors.

## 2 Study participants and methods

### 2.1 Study participants

This study was a single-center prospective longitudinal observational study. Using a consecutive sampling method, patients hospitalized with acute stroke were consecutively screened at the Department of Neurology, the Sixth People’s Hospital of Nantong, from February 2023 to October 2025. (1) Inclusion criteria: ① Age ≥ 18 years; ② Diagnosed with first-ever acute ischemic stroke or intracerebral hemorrhage confirmed by head CT or MRI; ③ Time from symptom onset to hospital admission ≤ 14 days; ④ Clear consciousness, with a Glasgow Coma Score (GCS) ≥ 13, or judged by a physician to possess basic communication ability; ⑤ Informed consent provided, voluntarily participating in the study and signing the informed consent form. (2) Exclusion criteria: ① Transient ischemic attack, subarachnoid hemorrhage, or other non-stroke neurological diseases; ② Presence of severe aphasia, sensory aphasia, severe cognitive impairment, or a history of dementia, preventing completion of scale assessments; ③ Accompanied by severe comorbidities with an expected survival of less than 6 months; ④ Inability to communicate effectively due to mental illness, deafness, or other reasons.

As the key analysis of this study was to identify latent perception trajectory classes, the specific number of which was unknown prior to data analysis, a precise *a priori* sample size calculation based on inter-group comparisons was not feasible. Estimation was conducted following general principles for Latent Class Growth Analysis and longitudinal studies (10, 11). Since the key analysis of this study was to identify latent trajectory classes of risk perception, the exact number of classes was unknown prior to data analysis, making it impossible to perform a precise *a priori* power calculation based on between-group comparisons. According to methodological recommendations for Latent Class Growth Analysis (LCGA), a total sample size of at least 200 participants is generally recommended to ensure model stability and class identifiability. In addition, considering an expected attrition rate of approximately 20% over the six-month follow-up period, this study planned to enroll no fewer than 250 patients. Ultimately, 262 patients were enrolled and 240 completed the study, meeting the basic requirements for LCGA and providing sufficient statistical power for subsequent comparisons of clinical outcomes across trajectory groups. This study was approved by the Ethics Committee of Nantong Sixth People’s Hospital (Approval No.: NTLYLL2023007). All participants provided written informed consent.

### 2.2 Research instruments and outcome measures

This study employed standardized questionnaires and clinical assessment tools for data collection. All scales were administered by uniformly trained researchers to patients via face-to-face assessment at designated time points.

#### 2.2.1 General information questionnaire

A self-designed questionnaire was used to collect sociodemographic data, including age, gender, marital status, education level, living situation, and mode of healthcare payment. Clinical data were extracted from the electronic medical record system, encompassing stroke type, time from onset to hospital admission, lesion location, past medical history, and the National Institutes of Health Stroke Scale score at admission.

#### 2.2.2 Stroke recurrence risk perception in patients

The Stroke Recurrence Risk Perception Scale, developed by Lin et al. (12) in 2022, was used for measurement. This scale consists of two parts with a total of 20 items. Part I (Perceived Likelihood of Recurrence): Contains 3 items. Item 1 assesses overall risk using a 1–5 point scale (“no risk” to “high risk”). Items 2–3 assess the perceived likelihood of recurrence under specific scenarios using a 0–10 point visual analog scale (“completely impossible” to “definitely will recur”). If “do not know” or “no risk” is selected for Item 1, Items 2–3 are not answered and are directly scored as 0. The total score for Part I ranges from 1 to 25 points. Part II: Contains three dimensions with a total of 17 items, using a 1–3 point scale (1 = disagree, 2 = uncertain, 3 = agree). The dimensions are: Perceived Severity of Recurrence, Perceived Disease-Related Risk Factors for Recurrence, and Perceived Behavioral Risk Factors for Recurrence. The total score for Part II ranges from 17 to 51 points. The sum of scores from Part I and Part II, ranging from 18 to 76 points. A higher score indicates a higher level of perceived disease recurrence risk in the patient. The overall Cronbach’s α coefficient for Part II of this scale is 0.850, with coefficients for each dimension ranging from 0.804 to 0.875, indicating good reliability. In this study, this scale was used for dynamic assessment at four time points: before hospital discharge (T0), and 1 month (T1), 3 months (T2), and 6 months (T3) after discharge, in order to construct the risk perception trajectories.

#### 2.2.3 Outcome measures

1. Neurological recovery and disability level.

Assessed using the Modified Rankin Scale (mRS). This scale is an internationally recognized core tool for measuring functional recovery and the degree of disability after stroke, with a scoring range of 0–6 points (0 = no symptoms, 6 = death) (13). The mRS score at 6 months served as a primary outcome variable in this study and was analyzed as an ordinal variable. Concurrently, for the purpose of binary logistic regression analysis, mRS scores were dichotomized into “good prognosis” (0–2 points, indicating functional independence) and “poor prognosis” (3–5 points, indicating functional dependence).

1. Neurological deficit severity assessment.

Assessed using the National Institutes of Health Stroke Scale (NIHSS). This is currently the most widely used and authoritative standardized tool globally for evaluating neurological deficits in stroke (14). Developed by Brott et al. (15) in 1989, the NIHSS comprises 11 examination items assessing the level of consciousness and orientation, gaze, visual fields, facial palsy, motor function of the upper and lower limbs, limb ataxia, sensation, language, dysarthria, and neglect. Each item has clear scoring criteria, assigning points from 0 (normal or no deficit) to 2, 3, or 4 points (severe deficit) based on the severity of the neurological impairment. The total scale score ranges from 0 to 42, with a higher score indicating more severe neurological deficits.

1. Activities of daily living ability.

Assessed using the Barthel Index (BI) (16). This scale evaluates a patient’s independence in 10 basic activities of daily living, such as feeding, bathing, dressing, and toileting. The total score ranges from 0 to 100, with a higher score indicating greater independence.

1. Stroke-specific quality of life assessment.

Assessed using the Stroke-Specific Quality of Life Scale (SS-QOL). This is an internationally widely used, disease-specific quality of life assessment tool designed for stroke patients, capable of comprehensively and sensitively reflecting multiple aspects of health-related quality of life after stroke. Developed by Williams et al. (17) in 1999, the Chinese version has been widely used domestically and possesses good psychometric properties. The assessment was conducted via patient self-report, completed under the guidance of researchers. Each item uses a 5-point Likert scoring method. The scale contains a total of 49 items belonging to 12 domains, with a total score range from 49 to 245 points. A higher total score indicates better stroke-specific quality of life.

1. Stroke recurrence event.

During the 6-month follow-up period, all new symptomatic stroke events confirmed by head CT or MRI were recorded through regular outpatient reviews, telephone follow-ups, and medical record system checks. These events were adjudicated by a neurologist, blinded to the patients’ risk perception data, based on clinical and imaging evidence.

### 2.3 Data collection methods

This study employed a prospective longitudinal design for data collection, with time points including baseline (T0, before discharge), and 1 month (T1), 3 months (T2), and 6 months (T3) after discharge. The research team consisted of uniformly and standardized-trained research coordinators and neurological assessors, with assessor blinding implemented. At the T0 time point, a research coordinator completed the collection of sociodemographic and clinical data. Assessor A (blinded to the patient’s subsequent risk perception data) then conducted the baseline assessment using the Modified Rankin Scale, the National Institutes of Health Stroke Scale, the Barthel Index, the Stroke-Specific Quality of Life Scale. Following this, the research coordinator guided the patient in completing the first assessment using the Stroke Recurrence Risk Perception Scale.

At the T1 and T2 time points, the research coordinator conducted repeated assessments of the recurrence risk perception scale via telephone or outpatient follow-up. At the T3 endpoint follow-up, Assessor B (blinded to all of the patient’s previous risk perception scores) repeated the assessment of all clinical outcome scales. Simultaneously, the research coordinator conducted the fourth risk perception assessment. Additionally, at the T3 time point, a neurologist (blinded to the study) reviewed all medical records to confirm stroke recurrence events.

To ensure data quality and minimize attrition, a “multi-channel contact” strategy was employed, and dual independent data entry and verification were performed. All assessment procedures adhered to standard operating protocols.

### 2.4 Statistical analysis

Data analysis was performed using SPSS 26.0 and Mplus 8.3 software. First, normality tests were conducted for continuous variables. Normally distributed data were described as mean ± standard deviation, and between-group comparisons were performed using independent samples *t*-test or analysis of variance (ANOVA). Non-normally distributed data were described as median and interquartile range, and non-parametric tests were used. Categorical variables were described as frequencies and percentages, and compared using the chi-square test or Fisher’s exact test. Second, LCGA was employed to identify longitudinal trajectories of disease recurrence risk perception. Using the total scores of the perception scale at the four time points as manifest variables, an unconditional LCGM was constructed. The number of latent classes was increased incrementally starting from one class. Model fit indices, including the Bayesian Information Criterion (BIC), Akaike Information Criterion (AIC), the significance of likelihood ratio tests, and entropy, were compared to determine the optimal number and shape of trajectory classes. Subsequently, the identified trajectory classes were used as grouping variables to compare differences in demographic and clinical baseline characteristics across trajectory groups. Finally, to examine the associations between trajectory classes and six-month clinical outcomes, multiple linear regression and multiple ordinal/binary logistic regression analyses were performed after controlling for potential confounders including age, sex, baseline NIHSS score, and stroke type. All statistical tests were two-sided, and a *p* value of less than 0.05 was considered statistically significant.

## 3 Results

### 3.1 Baseline characteristics of the study participants

A total of 262 patients met the eligibility criteria and agreed to participate in the study, all of whom provided written informed consent. During the six-month follow-up period, 22 patients were lost to follow-up (attrition rate: 8.4%), including 12 who could not be contacted due to invalid contact information, 7 who voluntarily withdrew, and 3 who relocated to other cities and were unable to complete the follow-up assessments. Consequently, a total of 240 patients completed the baseline and all four follow-up assessments and were included in the final data analysis, yielding a valid analysis rate of 91.6%. No statistically significant differences were observed in baseline characteristics between the completers and those lost to follow-up (all *p* > 0.05; see Supplementary Table S1). The baseline characteristics of the 240 acute stroke patients included in the final analysis are presented in Table 1. Among them, 148 (61.7%) were male and 92 (38.3%) were female. The age of the patients ranged from 36 to 88 years, with a mean age of 65.3 ± 10.8 years shown in Table 1.

**Table 1:** Baseline characteristics of stroke patients (*N* = 240).

Characteristic	Category	Number (*N*)	Percentage (%)
Gender	Male	148	61.7
	Female	92	38.3
Marital status	Married	215	89.6
	Unmarried/Divorced/Widowed	25	10.4
Education level	Junior high school or below	168	70.0
	High school or technical secondary school	52	21.7
	College or above	20	8.3
Health insurance	Urban Employee Basic Medical Insurance	182	75.8
	Urban and Rural Resident Basic Medical Insurance	55	22.9
	Self-pay/Other	3	1.3
Stroke type	Ischemic Stroke	221	92.1
	Hemorrhagic Stroke	19	7.9
TOAST classification (ischemic stroke, *n* = 221)	Large Artery Atherosclerosis	89	40.2
	Cardioembolism	45	20.4
	Small Vessel Occlusion	87	39.4
Major comorbidities	Hypertension	180	75.0
	Diabetes	85	35.4
	Hyperlipidemia	102	42.5
	Atrial Fibrillation	38	15.8
Baseline NIHSS score (points, median [IQR])	—	3 [1, 6]	—

### 3.2 Characteristics of trajectories for disease recurrence risk perception

Based on the total scores from the Stroke Recurrence Risk Perception Scale across four time points, this study employed LCGA to identify the heterogeneous developmental trajectories of patients’ risk perception over a 6-month period. The model fit indices are presented in Table 2. As the number of trajectory classes increased from 1 to 5, theBIC and AIC values continued to decrease, and the entropy values were all greater than 0.8. When the number of classes was increased to 4, the result of the LRT became statistically significant, and the average posterior probabilities for all classes exceeded 0.85, indicating good classification accuracy. Although the five-class model yielded slightly lower AIC and BIC values, the smallest class in the five-class solution accounted for only 10.0% (24/240) of the sample, and its clinical characteristics were difficult to distinguish from the “gradual declining perception” group in the four-class model. Taking into account the significance of the Lo–Mendell–Rubin likelihood ratio test (LMR-LRT), entropy values (0.89 for the four-class model vs. 0.85 for the five-class model), the minimum class proportion (>5%), and clinical interpretability, the four-class model was ultimately selected as the optimal solution.

**Table 2:** Model fit information for latent class growth analysis of stroke patients’ recurrence risk perception trajectories.

Model	AIC	BIC	Entropy	LMR-LRT *p* value	Class Probabilities (%)
1-Class	5421.36	5442.18	—	—	100
2-Class	5189.74	5220.11	0.821	<0.001	68.3/31.7
3-Class	5088.45	5128.37	0.845	0.012	41.2/35.4/23.4
4-Class	5023.71	5073.18	0.872	0.038	35.0/28.3/20.8/15.9
5-Class	5001.25	5060.27	0.886	0.214	26.7/20.0/28.3/15.0/10.0

AIC, Akaike Information Criterion; BIC, Bayesian Information Criterion; LMR-LRT, Lo–Mendell–Rubin Likelihood Ratio Test.

The four identified latent trajectory classes are shown in Figure 1.

**Figure 1:**
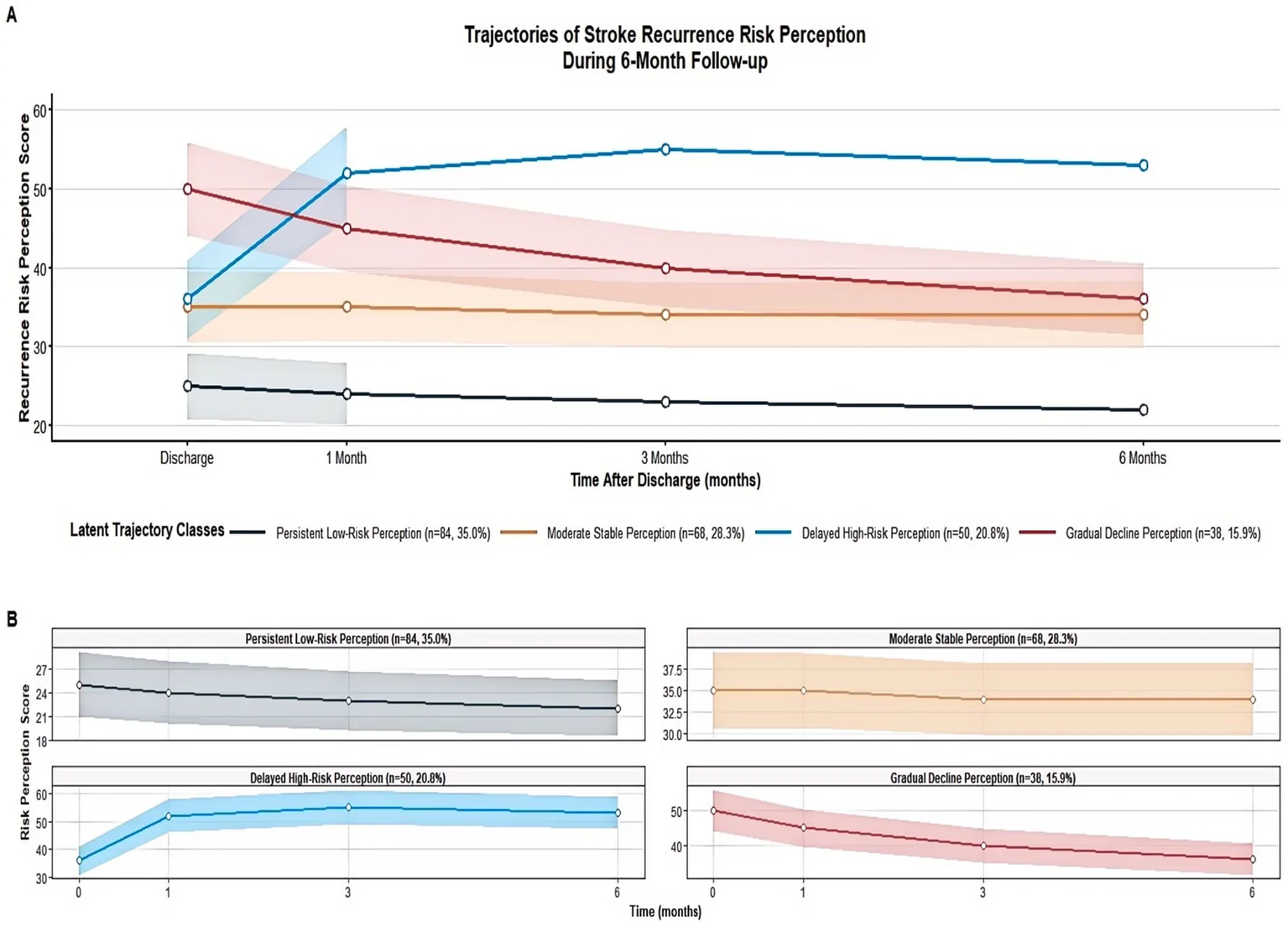
**(A)** Combined plot of the four latent trajectory classes: Persistent Low-Risk Perception (*n* = 84, 35.0%), Moderate Stable Perception (*n* = 68, 28.3%), Delayed High-Risk Perception (*n* = 50, 20.8%), and Gradual Decline Perception (*n* = 38, 15.9%). Shaded areas represent the 95% confidence intervals. **(B)** Separate subplots for each of the four latent trajectory classes.

Persistent low-risk perception group (35.0%, *n* = 84): Patients in this group had the lowest level of risk perception among the four groups at baseline (T0). This level remained stable throughout the 6-month follow-up period, showing a slight declining trend.

Moderate stable perception group (28.3%, *n* = 68): Patients in this group had a moderate level of risk perception at baseline. Their trajectory was the flattest over the entire follow-up period, with minimal fluctuation, presenting a stable state.

Delayed high-risk perception group (20.8%, *n* = 50): Patients in this group had a moderate level of risk perception at baseline. However, their perception level increased sharply and significantly within 1 month after discharge (T1) and remained persistently high during the T2 to T3 period. Although it slightly decreased by the T3 time point, it remained the highest among the four groups.

Gradual decline perception group (15.9%, *n* = 38): Patients in this group exhibited the highest level of risk perception at baseline, but showed a continuous, slow linear declining trend over time after discharge.

### 3.3 Comparison of baseline characteristics across trajectory groups

No statistically significant differences were observed in baseline demographic or clinical characteristics among the four trajectory groups (all *p* > 0.05). However, baseline total scores of recurrence risk perception differed significantly across the four groups (*p* < 0.001), as shown in Table 3.

**Table 3:** Comparison of baseline characteristics across different risk perception trajectory groups.

Variable	Category	Persistent low-risk group (*n* = 84)	Moderate stable group (*n* = 68)	Delayed high-risk group (*n* = 50)	Gradual decline group (*n* = 38)	Statistic	*p* value
Age (years, mean ± SD)		64.2 ± 10.3	65.8 ± 11.2	66.1 ± 10.5	63.9 ± 9.8	*F* = 0.52	0.669
Sex (*n*, %)	Male	53 (63.1)	42 (61.8)	33 (66.0)	20 (52.6)	χ^2^ = 1.81	0.613
	Female	31 (36.9)	26 (38.2)	17 (34.0)	18 (47.4)		
Marital status (*n*, %)	Married	76 (90.5)	60 (88.2)	43 (86.0)	36 (94.7)	χ^2^ = 1.93	0.587
	Unmarried/divorced/widowed	8 (9.5)	8 (11.8)	7 (14.0)	2 (5.3)		
Education level (*n*, %)	Junior high school or below	60 (71.4)	46 (67.6)	37 (74.0)	25 (65.8)	χ^2^ = 1.02	0.796
	High school or technical secondary school	17 (20.2)	16 (23.5)	10 (20.0)	9 (23.7)		
	College degree or above	7 (8.3)	6 (8.8)	3 (6.0)	4 (10.5)		
Medical payment method (*n*, %)	Urban employee basic medical insurance	65 (77.4)	50 (73.5)	39 (78.0)	28 (73.7)	χ^2^ = 0.51	0.917
	Urban/rural resident basic medical insurance	18 (21.4)	17 (25.0)	10 (20.0)	10 (26.3)		
	Self-paid or others	1 (1.2)	1 (1.5)	1 (2.0)	0 (0.0)		
Stroke type (*n*, %)	Ischemic	78 (92.9)	62 (91.2)	45 (90.0)	36 (94.7)	χ^2^ = 0.88	0.830
	Hemorrhagic	6 (7.1)	6 (8.8)	5 (10.0)	2 (5.3)		
TOAST classification (ischemic patients only) (*n*, %)*	Large artery atherosclerosis	32 (41.0)	25 (40.3)	18 (40.0)	14 (38.9)	χ^2^ = 0.04	0.998
	Cardioembolism	15 (19.2)	13 (21.0)	9 (20.0)	8 (22.2)		
	Small artery occlusion	31 (39.7)	24 (38.7)	18 (40.0)	14 (38.9)		
Comorbidities (*n*, %)	Hypertension	64 (76.2)	50 (73.5)	38 (76.0)	28 (73.7)	χ^2^ = 0.19	0.978
	Diabetes mellitus	31 (36.9)	23 (33.8)	18 (36.0)	13 (34.2)	χ^2^ = 0.17	0.982
	Hyperlipidemia	37 (44.0)	28 (41.2)	20 (40.0)	17 (44.7)	χ^2^ = 0.34	0.951
	Atrial fibrillation	14 (16.7)	10 (14.7)	8 (16.0)	6 (15.8)	χ^2^ = 0.11	0.950
Baseline NIHSS score (points, median [Q1, Q3])		3 [1, 5]	3 [1, 5]	4 [2, 7]	3 [1, 5]	H = 5.83	0.120
Total recurrence risk perception score at T0 (points, mean ± SD)		25.2 ± 3.8	35.1 ± 4.2	36.4 ± 4.5	50.3 ± 5.1	*F* = 215.46	<0.001

^*^Percentages for TOAST classification were calculated based on ischemic stroke patients within each group. Continuous variables were compared using one-way ANOVA (F value); non-normally distributed data were compared using the Kruskal–Wallis test (H value); categorical variables were compared using the chi-square test (χ^2^).

### 3.4 Univariate analysis of the impact of different risk perception trajectories on short-term clinical outcomes

To preliminarily examine the associations between different risk perception trajectories and six-month clinical outcomes, differences in outcomes across trajectory groups before adjusting for confounders were compared, as shown in Table 4 and Figure 2. Univariate analysis revealed significant differences among the four trajectory groups in mRS functional status distribution, mRS score, NIHSS score, NIHSS grade, BI score, BI independence grade, SS-QOL total score, and stroke recurrence rate (all *p* < 0.05). Among these, the delayed high-risk perception group demonstrated the strongest associations with the worst clinical outcomes across multiple indicators.

**Table 4:** Univariate analysis of clinical outcomes at 6 months across different risk perception trajectory groups.

Outcome measure	Persistent low-risk group (*n* = 84)	Moderate stable group (*n* = 68)	Delayed high-risk group (*n* = 50)	Gradual decline group (*n* = 38)	Statistic	*p*
Modified Rankin Scale (mRS)						
Functional status, *n* (%)					χ^2^ = 22.15	<0.001
0–2 points (Independent)	65 (77.4)	45 (66.2)	18 (36.0)	26 (68.4)		
3–5 points (Dependent)	19 (22.6)	23 (33.8)	32 (64.0)	12 (31.6)		
Score, median [IQR]	1 [1, 2]	2 [1, 3]	3 [2, 4]	2 [1, 3]	H = 23.42	<0.001
NIHSS						
Score, median [IQR]	1 [0, 3]	2 [1, 4]	4 [2, 7]	2 [1, 4]	H = 28.67	<0.001
Deficit severity, *n* (%)					χ^2^ = 18.93	<0.001
Normal/near normal (0–1)	48 (57.1)	32 (47.1)	10 (20.0)	18 (47.4)		
Mild deficit (2–4)	28 (33.3)	25 (36.8)	22 (44.0)	15 (39.5)		
Moderate/severe deficit (≥5)	8 (9.5)	11 (16.2)	18 (36.0)	5 (13.2)		
Barthel index (BI)						
Score, mean ± SD	88.5 ± 15.2	81.3 ± 18.6	70.4 ± 20.8	83.8 ± 16.9	*F* = 12.85	<0.001
Independence level, *n* (%)					χ^2^ = 13.04	0.005
Complete independence (100)	20 (23.8)	12 (17.6)	5 (10.0)	8 (21.1)		
Mild dependence (≥95)	52 (61.9)	34 (50.0)	15 (30.0)	20 (52.6)		
Moderate/severe dependence (<95)	12 (14.3)	22 (32.4)	30 (60.0)	10 (26.3)		
SS-QOL						
Total score, mean ± SD	205.3 ± 28.4	196.2 ± 31.5	175.8 ± 35.1	194.8 ± 29.7	*F* = 11.92	<0.001
Stroke recurrence						
Yes, *n* (%)	4 (4.8)	5 (7.4)	9 (18.0)	2 (5.3)	χ^2^ = 8.48	0.037

Continuous variables conforming to a normal distribution (BI, SS-QOL total score) are presented as mean ± standard deviation (Mean ± SD), with inter-group comparisons conducted using one-way ANOVA, reporting the F statistic. Non-normally distributed variables (mRS, NIHSS scores) are presented as median [interquartile range] (Median [IQR]), with inter-group comparisons conducted using the Kruskal–Wallis H test, reporting the H statistic. Categorical variables are presented as number (percentage), with inter-group comparisons conducted using the Chi-square test, reporting the χ^2^ statistic. Since stroke recurrence events were few (*n* = 20), this outcome was not analyzed in multivariable models; recurrence findings are exploratory and descriptive only.

**Figure 2:**
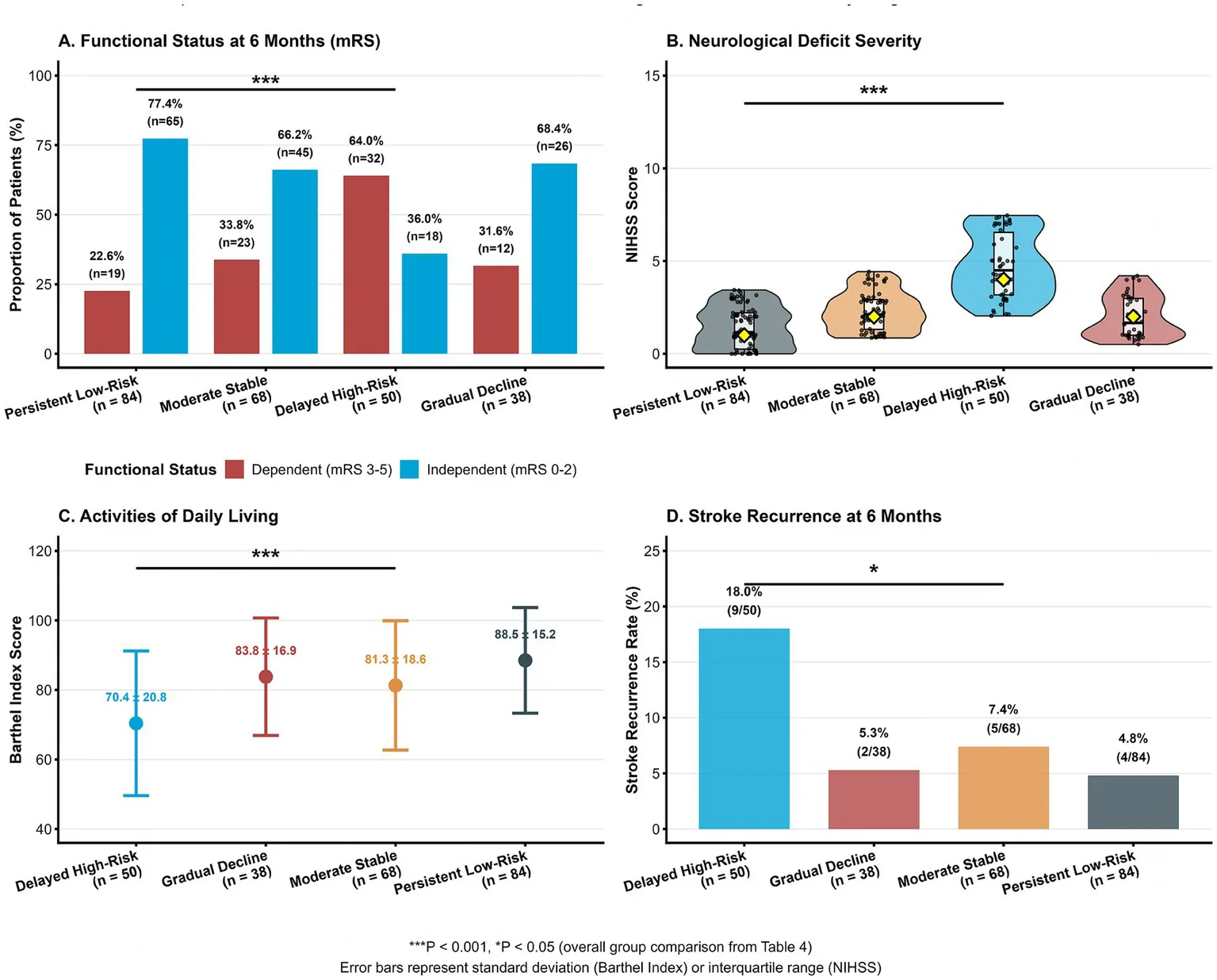
**(A)** Functional status at 6 months (mRS); **(B)** Neurological deficit severity (NIHSS score); **(C)** Activities of daily living (Barthel Index score); **(D)** Stroke recurrence rate at 6 months.

### 3.5 Multivariate regression analysis of the impact of different risk perception trajectories on clinical outcomes

To examine the independent associations between different risk perception trajectories and 6-month clinical outcomes, multivariable regression analyses were performed with adjustment for covariates including age, sex, marital status, education level, medical payment method, stroke type, baseline NIHSS score, hypertension, diabetes mellitus, hyperlipidemia, and atrial fibrillation. All models used the Persistent Low-Risk Perception Group as the reference. The results (Tables 5, 6) showed that the Delayed High-Risk Perception trajectory was significantly associated with most adverse clinical outcomes.

**Table 5:** Logistic regression results for binary outcomes (functional dependence).

Outcome variable	Trajectory group	aOR	95% CI	Wald χ^2^	*p* value
Functional dependence (mRS 3–5)	Moderate stable	1.30	0.61–2.78	0.68	0.496
	Delayed high-risk	2.85	1.35–6.01	7.54	0.006
	Gradual declining	1.55	0.67–3.58	1.06	0.304
Model fit: Nagelkerke R^2^ = 0.278, Hosmer-Lemeshow test *p* = 0.412, Model χ^2^(16) = 35.24, *p* < 0.001					

All multivariable models controlled for the following covariates: age, sex, marital status, education level, medical payment method, stroke type, baseline NIHSS score, hypertension, diabetes, hyperlipidemia, and atrial fibrillation.

**Table 6:** Linear regression results for continuous outcomes (NIHSS, BI, SS-QOL).

Outcome variable	Trajectory group	*β*	95% CI	*t* value	*p* value
NIHSS score	Moderate stable	0.61	−0.70 – 1.92	0.87	0.386
	Delayed high-risk	2.01	0.67–3.35	8.73	0.003
	Gradual declining	0.72	−0.60 – 2.04	1.13	0.291
Model fit: Adjusted R^2^ = 0.203, F(16,223) = 4.55, *p* < 0.001					
Barthel index	Moderate stable	−4.52	−11.77 – 2.73	1.51	0.220
	Delayed high-risk	−12.31	−20.13 – −4.49	9.57	0.002
	Gradual declining	−4.01	−12.38 – 4.36	0.90	0.345
Model fit: Adjusted R^2^ = 0.186, F(16,223) = 4.20, *p* < 0.001					
SS-QOL total score	Moderate stable	−3.81	−11.33 – 3.71	0.99	0.318
	Delayed high-risk	−14.61	−22.93 – −6.29	11.94	0.001
	Gradual declining	−5.90	−15.04 – 3.24	1.63	0.202
Model fit: Adjusted R^2^ = 0.194, F(16,223) = 4.41, *p* < 0.001					

All multivariable models controlled for the following covariates: age, sex, marital status, education level, medical payment method, stroke type, baseline NIHSS score, hypertension, diabetes, hyperlipidemia, and atrial fibrillation.

Specifically, regarding functional dependence: patients in the Delayed High-Risk Perception Group had an odds ratio of 2.85 for functional dependence (mRS 3–5) at 6 months compared with the reference group (aOR = 2.85, 95% CI: 1.35–6.01, *p* = 0.006), and the overall logistic regression model was statistically significant (*p* < 0.001). Regarding neurological impairment: the NIHSS score in the Delayed High-Risk Perception Group was on average 2.01 points higher than that in the reference group (95% CI: 0.67–3.35, *p* = 0.003). Regarding daily living ability: the Barthel Index score in this group was on average 12.31 points lower than that in the reference group (95% CI: −20.13 to −4.49, *p* = 0.002). Regarding quality of life: the SS-QOL total score in this group was on average 14.61 points lower than that in the reference group (95% CI: −22.93 to −6.29, *p* = 0.001). The overall linear regression models were all statistically significant (all *p* < 0.001).

Regarding stroke recurrence, a total of 20 recurrence events occurred during the 6-month follow-up period. The recurrence rates were 4.8% (*n* = 4) in the Persistent Low-Risk Perception Group, 7.4% (*n* = 5) in the Moderate Stable Perception Group, 18.0% (*n* = 9) in the Delayed High-Risk Perception Group, and 5.3% (*n* = 2) in the Gradual Decline Perception Group, with a statistically significant difference among groups (χ^2^ = 8.48, *p* = 0.037; Table 3). Given the small total number of recurrence events (*n* = 20), which was insufficient to support robust multivariable regression modeling, no multivariable-adjusted analysis was performed for the stroke recurrence outcome, and this finding is presented only as an exploratory descriptive result.

The Moderate Stable Perception and Gradual Decline Perception trajectories showed no statistically significant associations with any outcome in the multivariable analyses (all *p* > 0.05) (see Figure 3).

**Figure 3:**
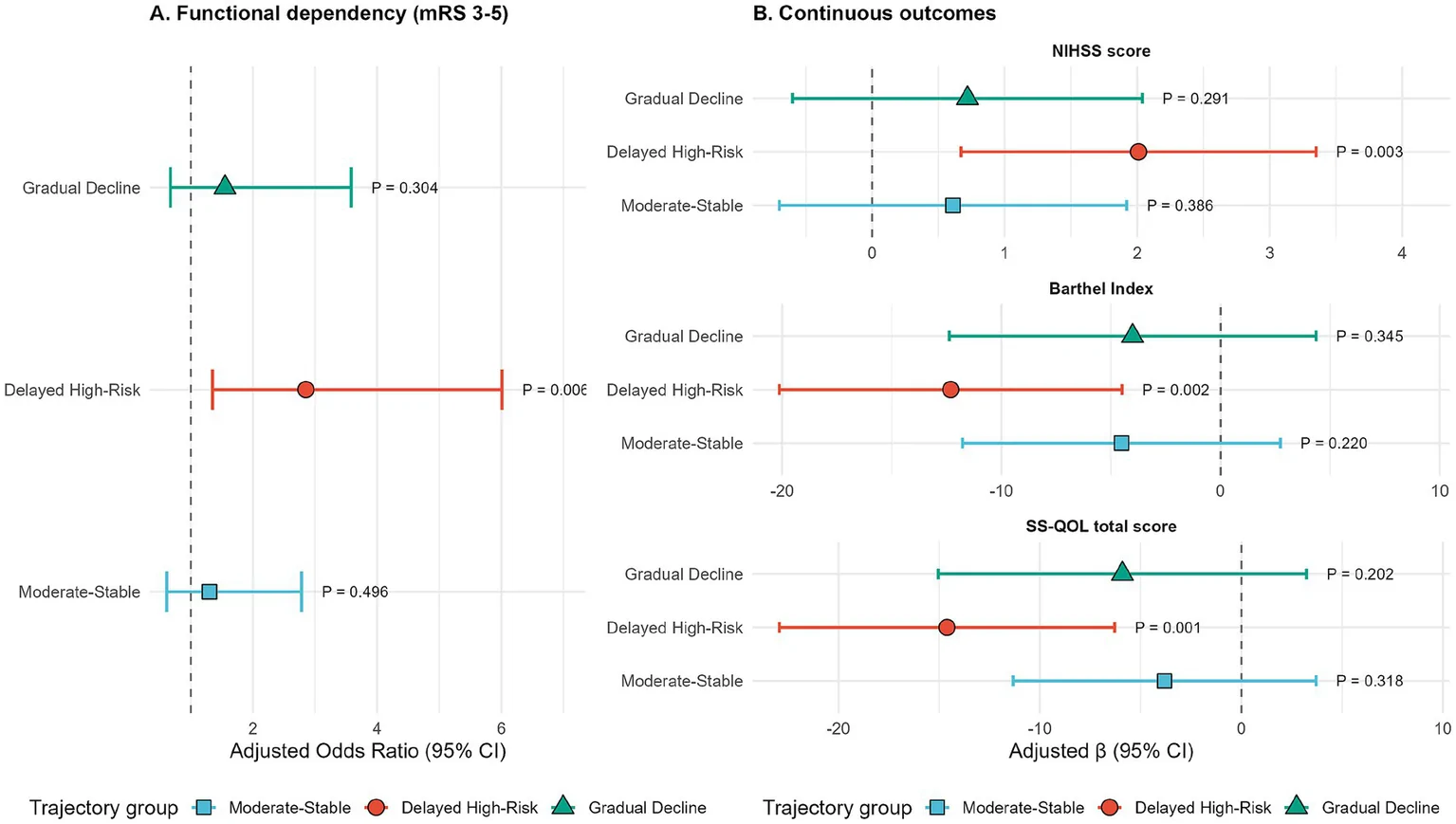
**(A)** Adjusted Odds Ratio (95% CI) for functional dependency (mRS 3-5); **(B)** Adjusted β coefficients (95% CI) for continuous outcomes (NIHSS score, Barthel Index, and SS-QOL total score).

### 3.6 E-value assessment for unmeasured confounding

To quantitatively assess the potential impact of unmeasured confounding on the main findings of this study, an E-value analysis was performed. The E-value is a sensitivity analysis metric that quantifies the minimum strength of association that an unmeasured confounder would need to have with both the exposure and the outcome to explain away or overturn the observed exposure–outcome association (18). It is calculated based on the effect estimate and its confidence interval after adjusting for known confounders. A larger E-value indicates greater robustness of the study findings to potential unmeasured confounding; conversely, a smaller E-value suggests that the results could be more easily explained away by a relatively weak unmeasured confounder.

In this study, using the adjusted odds ratios or regression coefficients obtained from multivariable regression analyses (approximated to risk ratios as the effect measure), E-values were calculated for the associations between the “Delayed High-Risk Perception” trajectory (with the “Persistent Low-Risk Perception” trajectory as the reference) and each key clinical outcome. For binary outcomes (functional dependence, stroke recurrence), E-values were computed directly based on the observed OR and its 95% confidence interval. For continuous outcomes (NIHSS, BI, SS-QOL), because the E-value is defined on the risk ratio scale, the linear regression coefficient (β) was converted to an approximate risk ratio (aRR) using the formula: aRR = e^(β / SD), where SD is the population standard deviation of the corresponding outcome variable. This conversion is equivalent to standardizing the continuous outcome to unit standard deviation before regression, thereby interpreting β as the number of standard deviations change in the outcome per unit change in exposure, and subsequently transforming it to the risk ratio scale (19). Given the approximate nature of this conversion, the E-value results for continuous outcomes should be regarded as exploratory. Both the E-value for the point estimate and the E-value for the lower bound of the 95% confidence interval are reported; the latter provides the threshold of confounding required to overturn the association under the most conservative estimate. The results are presented in Table 7.

**Table 7:** E-value sensitivity analysis for the impact of unmeasured confounding.

Clinical outcome measure	Observed effect (delayed high-risk vs. persistent low-risk)	E-value (for point estimate)	E-value (for 95% CI lower limit)
Functional dependency (mRS 3–5)	aOR = 2.85 (1.35, 6.01)	4.10	1.99
Neurological deficit (NIHSS Score)	β = 2.01 (0.67, 3.35)	3.73	1.68
Activities of daily living (Barthel Index)	β = −12.31 (−20.13, −4.49)	7.25	2.91
Quality of life (SS-QOL Total Score)	β = −14.61 (−22.93, −6.29)	8.61	3.30

aOR, adjusted odds ratio; CI, confidence interval. For continuous outcomes, the β values were converted to approximate risk ratios for a one-standard-deviation change for E-value calculation.

For the primary outcome of functional dependence (aOR = 2.85), the E-value for the point estimate was 4.10, and the E-value for the lower confidence limit was 1.99. This indicates that to completely attribute the observed 185% increase in the odds of functional dependence associated with the Delayed High-Risk Perception trajectory to confounding (i.e., to shift the effect estimate to the null, aOR = 1), an unmeasured confounder would need to be associated with both the Delayed High-Risk Perception trajectory and functional dependence by a risk ratio of at least 4.10 (based on the point estimate) or 1.99 (based on the lower confidence limit). For all outcomes assessed, particularly the reductions in Barthel Index and quality of life, the E-values for the lower confidence limits ranged from 1.58 to 3.30. This means that to fully explain away the associations between the Delayed High-Risk Perception trajectory and these adverse outcomes, one would need to postulate a single unmeasured confounder independently associated with both the exposure and the outcome by a risk ratio of at least 1.58 to 3.30. In the context of stroke rehabilitation, known strong prognostic factors—such as extremely severe baseline neurological deficits, specific complications, or complete lack of social support—are typically associated with poor outcomes with risk ratios within or below this range. It should be emphasized that the E-value only quantifies the minimum strength required for an unmeasured confounder; it does not demonstrate that adjustment for measured covariates is adequate, nor does it substitute for testing the correctness of model specification.

## 4 Discussion

Through a six-month longitudinal design, this study systematically delineated the dynamic evolution trajectories of disease recurrence risk perception among stroke patients after discharge, and identified four significantly distinct trajectory subgroups: “Persistent Low-Risk Perception,” “Moderate Stable Perception,” “Gradual Declining Perception,” and “Delayed High-Risk Perception.” Multivariable regression analyses, after controlling for demographic and clinical characteristics, showed that, compared with the Persistent Low-Risk Perception group, the Delayed High-Risk Perception trajectory was significantly associated with functional dependence, worsened neurological deficits, reduced activities of daily living, and decreased quality of life at 6 months. These findings highlight, from a dynamic perspective, the close link between risk perception and stroke recovery outcomes, and suggest that early identification of and targeted intervention on patients’ psychological trajectories may hold important clinical implications.

The theoretical framework of this study aligns closely with the Health Belief Model and the Common-Sense Model of Illness Perceptions (20, 21). These theories emphasize that an individual’s cognitive appraisal of disease threat is central to driving their health behavior decisions. A cross-sectional study by Jin et al. (22) on stroke patients constructed a chain mediation model linking recurrence risk perception and health behaviors, confirming that behavioral decision-making plays a key mediating role in the process of how risk perception influences health behavior. The longitudinal data of this study provide empirical support for this theoretical pathway. More importantly, using latent class growth modeling, we confirmed that the dynamic pattern of risk perception itself, particularly the “Delayed High-Risk” trajectory characterized by an increase rather than a decrease after discharge—is an important psychological marker closely associated with poor prognosis. This finding differs from the study by Groeneveld et al. (23), which used cluster analysis to classify post-stroke illness perception trajectories into three categories (“favorable,” “average,” and “unfavorable”) and found that the “unfavorable” trajectory was associated with a higher risk of depressive symptoms at 12 months. Our study identified a more complex four-class structure, particularly the “Delayed High-Risk” group with the worst prognosis. This discrepancy may stem from differences in research focus (illness perception is a broader construct, whereas our study focused on the specific threat of recurrence risk), assessment instruments, follow-up density, or population heterogeneity, collectively suggesting that the psychological adaptation process among stroke patients exhibits substantial diversity and is not a single uniform pattern.

The potential mechanisms linking the “Delayed High-Risk Perception” trajectory to adverse outcomes may be multidimensional and interrelated. Possible explanations are hypothesized as follows. First, the abnormal elevation and sustained high level of risk perception after discharge may profoundly reflect patients’ loss of perceived control over their disease and deep concerns about future health (24); such psychological states may predispose to or exacerbate negative emotions such as anxiety and depression. Existing studies have confirmed that rehabilitation self-efficacy can influence family-based rehabilitation exercise adherence through its effects on recurrence risk perception and outcome expectations (25). Persistent negative emotional stress may, via activation of neuroendocrine pathways such as the hypothalamic–pituitary–adrenal axis, create a physiological environment unfavorable to neuroplasticity and repair (26). Second, according to fear control theory, when perceived threat far exceeds an individual’s perceived coping ability, maladaptive coping strategies may be triggered—such as avoidance of necessary rehabilitation exercises, non-adherence to medication regimens, or decision paralysis—rather than health-promoting protective behaviors (27, 28). It should be emphasized that these mechanistic pathways are primarily derived from theoretical reasoning and previous literature; this study did not directly measure neuroendocrine indicators or behavioral mediators, and therefore these explanations should be regarded as speculative mechanisms pending verification through mediation analyses or experimental designs in future research. In contrast, patients in the “Moderate Stable” and “Gradual Declining” trajectory groups may maintain a dynamic balance between risk perception and self-efficacy, which may be more conducive to sustaining treatment adherence and positive health behaviors.

The findings of this study offer certain clinical implications, although they should be interpreted with caution. The data suggest that clinicians may consider incorporating dynamic assessment of recurrence risk perception into routine follow-up management systems for stroke patients after discharge, rather than relying solely on a single assessment at admission (29). For patients identified early through trajectory analysis as belonging to the “Delayed High-Risk Perception” group, they could be considered a potential priority population for multidisciplinary psychological intervention. Intervention strategies may draw upon cognitive restructuring techniques based on cognitive behavioral therapy to help patients develop a more rational and controllable perception of disease recurrence risk, combined with coping skills training to enhance their practical abilities in problem-solving, stress management, and emotional regulation (30, 31). However, it must be clearly stated that this study adopted an observational design and did not test any intervention. Whether risk perception trajectories are modifiable, and whether modifying risk perception through intervention can improve clinical outcomes, remain open questions awaiting verification through future randomized controlled trials. When implementing interventions, a stratified management strategy targeting specific subgroups may be considered, for example, drawing on research on personalized stroke rehabilitation models, with particular attention to female patients, those with more severe baseline neurological deficits, or those with prominent anxiety symptoms—so as to achieve optimal allocation of healthcare resources.

This study has several limitations. First, this was a single-center study, and the representativeness of the sample may be subject to geographic constraints. Future multicenter studies with larger samples are needed to validate the generalizability of the trajectories and the stability of their predictive validity. Second, although we controlled for multiple known confounders through multivariable regression and conducted an E-value sensitivity analysis (indicating that the results were reasonably robust to unmeasured confounding), unmeasured confounding variables may still exist, such as the specific quality of social support networks, patients’ particular personality traits, or access to healthcare. Third, risk perception was primarily assessed using patient-reported scales, which may be subject to recall bias or social desirability bias. Future studies could incorporate objective physiological indicators or caregiver reports for multi-dimensional assessment. Fourth, the six-month follow-up period remains insufficient for observing endpoint events such as long-term stroke recurrence, rehospitalization, and chronic disability. Fifth, the analysis of stroke recurrence risk requires particular caution. Although the Delayed High-Risk Perception Group had the highest recurrence rate (18.0%), and the between-group difference in recurrence rates was statistically significant (*p* = 0.037), the total number of stroke recurrence events during the 6-month follow-up was small (only 20 cases), which did not meet the conventional assumptions for multivariable logistic regression and would render parameter estimates highly unstable. Therefore, no multivariable-adjusted analysis was performed for the stroke recurrence outcome in this study. Based on the available evidence, the between-group difference in recurrence rates should be regarded as an exploratory hypothesis rather than a confirmatory conclusion. Future studies with larger samples, particularly those with a greater number of recurrence events, are warranted to validate this association, and analytical methods suitable for sparse event data, such as Firth’s penalized likelihood or Bayesian logistic regression, could be considered to obtain more robust estimates. Sixth, there is also potential for reverse causation. That is, we cannot exclude the possibility that patients experienced an abnormally elevated level of risk perception after discharge due to poor neurological recovery or early complications, rather than risk perception itself leading to adverse outcomes. The Delayed High-Risk Perception trajectory may serve both as a psychological response to an unfavorable recovery process and as a factor that further impedes recovery. Future studies could adopt denser follow-up designs (e.g., weekly assessments) combined with analytical approaches such as cross-lagged panel models to better clarify the temporal relationships and bidirectional influences between risk perception and clinical outcomes. In this study, the construction of risk perception trajectories incorporated measurement data at T3 (6 months), while clinical outcomes were also assessed at the same time point. Therefore, there was temporal overlap between exposure and outcome at the endpoint, and our analysis essentially reflected concurrent associations rather than prospective prediction. Future studies could restrict trajectory construction to T0–T2 (from discharge to 3 months) to examine the predictive value of early risk perception changes for six-month outcomes and to more clearly delineate the temporal sequence.

Based on the above, future research could be pursued in the following directions: First, utilizing larger prospective cohorts to further explore antecedent variables influencing the formation of different risk perception trajectories, such as the quality of early rehabilitation experience, patterns of clinician–patient communication, and family and social support systems. Second, building on the four trajectories identified in this study, designing and implementing randomized controlled trials to verify the effectiveness and cost-effectiveness of targeted, individualized psychosocial intervention programs. Third, extending the follow-up period to 1 year or longer to clarify the predictive value of different risk perception trajectories for patients’ long-term functional independence, quality of life, and survival. Fourth, actively exploring the use of mobile health technologies to enable convenient, real-time, dynamic monitoring and immediate feedback on patients’ risk perception status, thereby providing a window of opportunity for early intervention.

## 5 Conclusion

This study confirms that heterogeneous longitudinal trajectories of disease recurrence risk perception exist among stroke patients. Among them, the “Delayed High-Risk Perception” trajectory characterized by a sharp increase in risk perception after discharge that remains persistently elevated was significantly associated with functional dependence, worsened neurological deficits, reduced activities of daily living, and decreased quality of life, serving as an important indicator for identifying adverse short-term clinical outcomes. The association between this trajectory and stroke recurrence risk requires further investigation. These findings suggest that dynamic monitoring of patients’ risk perception changes and attention to their psychological status in secondary stroke prevention may be clinically valuable, offering a new theoretical perspective and potential entry point for precision nursing practice.

## Data Availability

The original contributions presented in the study are included in the article/Supplementary material, further inquiries can be directed to the corresponding author.
