# Supplementary material for: A longitudinal study on the impact of stroke patients’ perception trajectories of disease recurrence on short-term clinical outcomes
Source: Front Neurol. 2026 Sep 25;17:1883000. doi: 10.3389/fneur.2026.1883000 (PMC13649446; doi:10.3389/fneur.2026.1883000)
Supplement: Supplementary file 1 [file Table_1.DOCX]

Supplementary Table S1 Comparison of Baseline Characteristics Between Completers and Non‑completers

| **Variable** | **Completers (n=240)** | **Non‑completers (n=22)** | **Statistic** | **P value** |
| --- | --- | --- | --- | --- |
| Age (years, mean ± SD) | 65.3 ± 10.8 | 64.1 ± 11.5 | t=0.49 | 0.624 |
| Male (n, %) | 148 (61.7) | 14 (63.6) | χ²=0.03 | 0.857 |
| Marital status (married, n, %) | 215 (89.6) | 20 (90.9) | Fisher | 1.000 |
| Education level (junior high school or below, n, %) | 168 (70.0) | 16 (72.7) | χ²=0.08 | 0.781 |
| Medical payment method (urban employee basic medical insurance, n, %) | 182 (75.8) | 17 (77.3) | χ²=0.00 | 0.987 |
| Stroke type (ischemic, n, %) | 221 (92.1) | 20 (90.9) | Fisher | 0.694 |
| TOAST classification (large artery atherosclerosis, n, %)* | 89 (40.3) | 8 (40.0) | χ²=0.01 | 0.976 |
| Hypertension (n, %) | 180 (75.0) | 16 (72.7) | χ²=0.09 | 0.764 |
| Diabetes (n, %) | 85 (35.4) | 8 (36.4) | χ²=0.01 | 0.925 |
| Hyperlipidemia (n, %) | 102 (42.5) | 9 (40.9) | χ²=0.02 | 0.885 |
| Atrial fibrillation (n, %) | 38 (15.8) | 3 (13.6) | Fisher | 1.000 |
| Baseline NIHSS score (points, median [Q1, Q3]) | 3 [1, 6] | 3 [2, 7] | Z=−0.46 | 0.645 |
| Baseline total recurrence risk perception score (points, mean ± SD) | 28.5 ± 9.2 | 29.1 ± 9.8 | t=−0.31 | 0.757 |

Note: Percentages for TOAST classification were calculated based on ischemic stroke patients (completers: n=221; non‑completers: n=20).
